# Quantifying radiation exposure in the radiological investigation of non-arthritic hip pain

**DOI:** 10.1093/jhps/hnae013

**Published:** 2024-04-01

**Authors:** Alistair IW Mayne, Ahmed Saad, Rajesh Botchu, Lucie Gosling, Peter Wall, Angelos Politis, Peter D’Alessandro, Callum McBryde

**Affiliations:** Orthopaedic Research Foundation of Western Australia, Perth, WA 6160, Australia; Orthopaedic Department, Fiona Stanley Fremantle Hospitals Group, Alma St, Fremantle, WA 6160, Australia; Royal Orthopaedic Hospital, Bristol Road South, Birmingham B31 2AP, UK; Royal Orthopaedic Hospital, Bristol Road South, Birmingham B31 2AP, UK; Royal Orthopaedic Hospital, Bristol Road South, Birmingham B31 2AP, UK; Royal Orthopaedic Hospital, Bristol Road South, Birmingham B31 2AP, UK; Royal Orthopaedic Hospital, Bristol Road South, Birmingham B31 2AP, UK; Royal Orthopaedic Hospital, Bristol Road South, Birmingham B31 2AP, UK; Orthopaedic Research Foundation of Western Australia, Perth, WA 6160, Australia; Orthopaedic Department, Fiona Stanley Fremantle Hospitals Group, Alma St, Fremantle, WA 6160, Australia; Medical School, Division of Surgery, University of Western Australia, Perth 6009, Western Australia; Royal Orthopaedic Hospital, Bristol Road South, Birmingham B31 2AP, UK

## Abstract

Radiological investigations are essential for evaluating underlying structural abnormalities in patients presenting with non-arthritic hip pain. The aim of this study is to quantify the radiation exposure associated with common radiological investigations performed in assessing patients presenting with non-arthritic hip pain. A retrospective review of our institutional imaging database was performed. Data were obtained for antero-posterior, cross-table lateral, frog lateral radiographs and low-dose CT hip protocol. The radiation dose of each imaging technique was measured in terms of dose-area product with units of mGy cm^2^, and the effective doses (ED, mSv) calculated. The effective radiation dose for each individual hip radiograph performed was in the range of 0.03–0.83 mSv [mean dose-area product 126.7–156.2 mGy cm^2^]. The mean ED associated with the low-dose CT hip protocol (including assessment of femoral anteversion and tibial torsion) was 3.04 mSv (416.8 mGy cm^2^). The radiation dose associated with the use of CT imaging was significantly greater than plain radiographs (*P* < 0.005). Investigation of non-arthritic hip pain can lead to significant ionizing radiation exposure for patients. In our institution, the routine protocol is to obtain an antero-posterior pelvic radiograph and then a specific hip sequence Magnetic Resonance Imaging (MRI) scan which includes the assessment of femoral anteversion. This provides the necessary information in the majority of cases, with CT scanning reserved for more complex cases where we feel there is a specific indication. We would encourage the hip preservation community to carefully consider and review the use of ionizing radiation investigations.

## INTRODUCTION

Non-arthritic hip pain can be a significant cause of pain and morbidity, particularly in the young adult population. There continues to be an increasing awareness of common treatable causes such as femoroacetabular impingement syndrome (FAIS) and hip dysplasia [1]. In addition to our increasing understanding of treatable pathologies, there has been significant innovation in surgical treatments in the management of these hip conditions, with many treatments now proven to lead to a significant improvement in patient symptoms and hip survivorship [2–5].

In patients with persisting or recurrent groin/hip pain, careful clinical evaluation by a clinician experienced in managing this patient population should be undertaken to obtain a diagnosis and allow management of treatable pathology. In establishing the aetiology of non-arthritic hip pain, radiological investigations are essential to allow detection of underlying structural abnormalities. However, the imaging modalities utilized within the workup of patients vary widely within the hip preservation community, influenced by both clinician preference and available resources. Common investigations include plain radiographs, CT and MRI.

Often, clinicians will also obtain routine post-operative radiological imaging. A recent international modified Delphi study to define the ‘acceptable’ surgical correction of FAIS concluded that surgical correction should be assessed intraoperatively with fluoroscopy and dynamic visual assessment, as well as with post-operative antero-posterior (AP) pelvis and Dunn lateral radiographs [6]. In this study, 27.5% of participants recommended obtaining a post-operative 45 degree Dunn lateral radiograph and 14.2% recommended a 3-dimensional (3D) CT scan. Among the expert panel, there was consensus (88%) that AP and Dunn lateral were the most important radiographs to evaluate the hip post-operatively.

Consequently, patients presenting with non-arthritic hip pain often undergo extensive radiological investigation, many of which involve ionizing radiation. Information quantifying the ionizing radiation exposure is important to consider when determining imaging modalities in the workup of the non-arthritic hip pain patient given the young age of many patients. There is evidence that many clinicians are poorly informed with regard to the radiation dose associated with investigations which they request [7, 8]. This information is important to enable an informed discussion with the patient so that they are appropriately informed and consented with regard to the amount of ionizing radiation as well as the potential risks associated with it. Additionally, improved awareness of the radiation exposure associated with imaging procedures can help surgeons rationalize their use of ionizing imaging in line with the principle of ALARA (as low as is reasonably achievable) [8].

The aim of this study is to quantify the radiation exposure associated with common radiological investigations performed in assessing young adult hip patients and to describe our local imaging protocol.

## METHODS

This study was performed at a tertiary hip preservation centre in the United Kingdom. A retrospective analysis was conducted using data retrieved from our computerized institutional radiology database to determine the mean effective dose (ED) to patients from different imaging protocols used in the workup of non-arthritic hip pain patients. The study included patients who had the following imaging performed: AP pelvis radiograph, cross-table lateral hip radiograph, frog lateral hip radiograph and a low-dose CT hip protocol.

Plain radiographs were performed using Philips Digital Diagnose Machines, a digital radiograph system. The comparison of radiation doses focused on AP pelvis radiographs, cross-table lateral, frog leg lateral, as well as CT images including the low-dose CT hip protocol (which included assessment of femoral anteversion and tibial torsion). All CT scans were performed on Siemens Somatom AS machines (Erlangen, Germany). The radiation dose of each imaging technique was measured in terms of dose-area product (DAP) using units of mGy cm^2^.

To determine the ED in mSv, the DAP values obtained from the imaging equipment were documented in our Computerized Radiology Information System database by the performing radiographer. These DAP values were then submitted to the Radiation Physicist for conversion using tables outlined in the International Commision on Radiological Protection 103 publication. The conversion process involved incorporating tissue weighting factors specified in the publication to assess the susceptibility of different tissues to cancer induction.

To allow calculation of lifetime increase in cancer risk, we used the validated online radiation risk assessment tool which was developed to allow estimation of the increased lifetime risk of radiation-related cancer (along with uncertainty intervals) following a known exposure history. The relative excess risk of developing cancer is the proportional increase in risk of cancer over the background absolute risk.

This service evaluation did not require ethical approval since all imaging studies were performed as part of routine patient care.

## RESULTS

We identified 18 plain AP pelvis radiographs, 13 cross-table lateral radiographs and 22 frog lateral radiographs for inclusion in the study, referred from the hip preservation service. Data from 21 patients undergoing a low-dose CT hip protocol scan were included. The ED associated with each radiographic investigation is documented in Table I, and that for each of the CT protocols is documented in Table II. Table III outlines the radiation dose for each investigation compared to commercial air travel and background radiation.

**Table I.** Radiation dose associated with plain radiographs

*Radiograph*	*Mean ED (mSv)*	*Mean DLP (mGy* *cm^2^)*	*Maximum DLP (mGy* *cm^2^)*	*Minimum DLP (mGy* *cm^2^)*	*Number of cases*
AP	0.022	126.7	433	48	18
Cross-table lateral	0.026	148.68	525.00	22.90	13
Frog lateral	0.027	156.18	301.00	13.00	22

**Table II.** Radiation dose associated with CT imaging

*CT scan*	*Mean ED (mSv)*	*Mean DLP (mGy* *cm^2^)*	*Maximum DLP (mGy* *cm^2^)*	*Minimum DLP (mGy* *cm^2^)*	*Number of cases*
Low-dose CT (hip protocol)	3.04	416.76	772	129	21

**Table III.** Comparison of radiation dose with each investigation in comparison to commercial air travel and background radiation exposure [24–26]

*Investigation*	*Mean ED (mSv)*	*Commercial air travel flight equivalent (hours)*	*Equivalent days of background radiation exposure*	*Relative excess risk of developing cancer*
Single AP radiograph	0.022	5.5	6.3	0.08 (0.02–0.18)
Single cross-table radiograph	0.026	6.5	7.4	0.09 (0.02–0.2)
Single frog lateral radiograph	0.027	6.75	7.7	0.1 (0.02–0.22)
Low-dose CT scan of hip including assessment of femoral anteversion	3.04	760	868.6	11.8 (2.9–25.1)

## DISCUSSION

This study has documented the ionizing radiation associated with different imaging modalities routinely used in the investigation of patients presenting with non-arthritic hip pain. In particular, the study has demonstrated the significant increase in ionizing radiation associated with CT imaging in comparison to plain radiographs.

Plain radiographs are generally the first-line investigation for any patient presenting with hip pain. It is the authors’ opinion that any young patient with persistent groin pain for >4 weeks or recurrent groin pain should undergo an AP pelvis radiograph, with early consideration for referral to a hip preservation specialist for specialist review and consideration of further imaging. A wide variety of radiographic views have been described to help clinicians to fully evaluate the complex 3D acetabular and proximal femoral anatomy, including AP pelvis, cross-table hip lateral, false-profile, frog lateral and 45-degree and 90-degree Dunn lateral, among others [9]. The radiographic views utilized vary widely between clinicians based on personal preferences and radiograph availability.

This study has demonstrated that plain radiographs expose patients to very low levels of ionizing radiation, with the mean ED 0.23 mSv for the three views included in the study. These views are comparable to the literature which documents the effective radiation dose for hip radiographs to be in the range 0.03–0.83 mSv [10, 11]. Although not noted in this study, the effective radiation dose has been shown to vary significantly between AP and lateral hip radiographic projections [11]. Positioning of patients for lateral radiographs can be challenging, especially in the absence of experienced radiographers, and this can increase the likelihood of repeat exposures. Young *et al*. reported that the ED for a cross-table lateral was significantly increased compared to other lateral hip radiographic projections (mean ED for cross-table lateral 0.83 mSv versus 0.37 mDv for Dunn lateral and 0.22 for single frog lateral) [11]. Based on this work, if a lateral radiograph is to be obtained, we suggest that a cross-table lateral should be avoided and preference should be given to alternative lateral radiographic projections such as the Dunn lateral or frog lateral.

Due to the complex anatomy of the hip, 3D imaging is almost universally obtained in the hip preservation community, frequently with CT imaging. CT imaging has traditionally been associated with much higher radiation exposure compared to conventional radiographs, although modern, low-dose protocols have been developed to reduce radiation exposure. This decrease in radiation exposure is largely attributable to improving technology in both hardware and software, with techniques such as iterative reconstruction significantly lowering radiation doses [10]. Additionally, because of the need to focus on the bony morphology as opposed to subtle changes in soft tissues, specific hip protocols can be developed to minimize the effective radiation dose [10]. Nonetheless, the mean ED for the low-dose CT hip protocol used in our institution is 3.04 mSv, which is significantly higher than the ED associated with plain radiographs. The literature has documented mean EDs of radiation for CT scans of the hip and pelvis in the range of 2.86–5.06 mSv [10, 12, 13]. This is comparable to the doses reported in the present study.

To put the radiation doses documented into context, the ED from background radiation is approximately 3 mSv per annum and a return flight from London to New York is 0.1 mSv [13]. Therefore, a single low-dose hip protocol CT scan is the equivalent of around 1 year of background radiation or 760 hours of commercial air travel.

Increasing use of ionizing radiation studies has led to concerns regarding the increased risk of radiation-induced malignancies for patients [14–16]. This is of particular concern for younger patients. Specific to the non-arthritic hip population, previous research has demonstrated a small increased risk of cancer, which is increased with repeated ionizing radiation investigations and younger patient age [10]. In a large population study of 680 000 patients undergoing CT imaging in childhood/adolescence, where the mean ED per scan was 4.5 mSv, the absolute excess cancer incidence was 9.3 per 100 000 person years at risk at a 10-year follow-up [14]. We have documented the relative excess risk of developing cancer with each investigation.

In our unit, the only routine ionizing radiation investigation used in the standard workup of a patient presenting with hip pain is a standing antero-posterior pelvic radiograph. This view will allow us to determine if the patient has significant osteoarthritis or significant chondral pathology or hip dysplasia or if there is large lateral cam morphology. Routinely using a radiographic calibration marker also ensures that if degenerative changes are shown on the plain film, repeating the imaging is not required. We do not believe that further plain radiographs are likely to significantly change our management plan. Therefore, if patients require further workup and evaluation, our next routine investigation is a 3 Tesla MRI scan with imaging of the pelvis, dedicated hip sequences, and T1 vibe axial imaging to allow calculation of femoral anteversion and tibial torsion. The imaging which patients have previously undergone is widely variable. The decision for whether to repeat the scan is made on a case-by-case basis, but is frequently obtained. We believe that the image quality on 3 Tesla hip-specific MRI sequences has greatly improved diagnostic quality compared to 1.5 Tesla scans (often performed with MRI arthrograms). We do not routinely obtain post-operative ionizing radiation imaging unless clinically indicated, for example, to assess bone healing following osteotomy surgery.

Assessment of femoral anteversion and tibial torsion has traditionally been performed via CT scan. However, with increasing advancements in MRI and development of specific rapid sequences such as T1 Volumetric Interpolated Breath-hold Examination (VIBE), MRI has now been shown to be comparable to CT in assessment of femoral version [17–21]. Previous research has also demonstrated that MRI has good agreement and comparable results in comparison to radiographs with regards to measurement of the lateral center-edge angle, Tönnis angle and extrusion index. However, the anterior centre-edge angle could not be reliably measured on MR in this study.

Because of the complex dynamic nature of FAIS, there has been increasing use of dynamic 3D simulation software in the investigation of hip impingement. The majority of commercially available packages currently use CT imaging, usually with thin slices and therefore potentially high ED protocols. Due to a reluctance to obtain routine CT scans for hip preservation patients due to the associated radiation, Lerch *et al*. investigated the potential use of MRI in developing 3D simulation models of hip impingement [22]. They reported that the difference between MRI and CT models was less than 1 mm for the acetabulum and proximal femur, and the correlation for range of motion values was excellent (*r* = 0.99, *P* < 0.001).

Consequently, MRI imaging can now provide the majority of the information required for the routine investigation of the non-arthritic hip pain patient. We would encourage commercial software to transition to MRI rather than CT to minimize radiation exposure to hip preservation patients. We acknowledge that CT continues to offer useful information in select cases, and therefore we reserve CT scanning for more complex cases where we feel that there is a specific indication.

It is important to note the limitations of this study. Quantifying the exact amount of radiation exposure that is received by patients is challenging due to the variations in patient size, complexity of radiation dose measurements and variable protocols between institutions. The ED therefore represents an estimate value taking into account the anatomic location, patient orientation, technique factors and published data converting exposure to whole-body ED [23]. Additionally, the number of patients undergoing lateral radiographs is small due to the fact that these images are not routinely used in our institution. Nonetheless, the mean values were in keeping with those published within the literature.

This study has quantified the radiation exposure associated with routine radiological investigation of patients presenting with non-arthritic hip pain. This information will help guide clinicians when considering investigations in the workup of these patients and allow surgeons to use imaging in line with the principle of ALARA [8]. We have described the routine imaging pathway used in our institution, which has minimal radiation exposure to the majority of patients. We would encourage the hip preservation community to carefully consider and review the routine use of higher-dose ionizing radiation investigations in the evaluation of young adults presenting with hip pain.

## Data Availability

The data underlying this article are available in the article and in its online supplementary material.
